# Preparation of a *β*-Cyclodextrin-Based Open-Tubular Capillary Electrochromatography Column and Application for Enantioseparations of Ten Basic Drugs

**DOI:** 10.1371/journal.pone.0146292

**Published:** 2016-01-15

**Authors:** Linlin Fang, Jia Yu, Zhen Jiang, Xingjie Guo

**Affiliations:** Department of Pharmaceutical Analysis, School of Pharmacy, Shenyang Pharmaceutical University, Ministry of Education, Shenyang, Liaoning Province, P. R. China; Hanyang University, REPUBLIC OF KOREA

## Abstract

An open-tubular capillary electrochromatography column was prepared by chemically immobilized *β*-cyclodextrin modified gold nanoparticles onto new surface with the prederivatization of (3-mercaptopropyl)-trimethoxysilane. The synthesized nanoparticles and the prepared column were characterized by transmission electron microscopy, scanning electron microscopy, infrared spectroscopy and ultraviolet visible spectroscopy. When the column was employed as the chiral stationary phase, no enantioselectivity was observed for ten model basic drugs. So *β*-cyclodextrin was added to the background electrolyte as chiral additive to expect a possible synergistic effect occurring and resulting in a better separation. Fortunately, significant improvement in enantioselectivity was obtained for ten pairs of drug enantiomers. Then, the effects of *β*-cyclodextrin concentration and background electrolyte pH on the chiral separation were investigated. With the developed separation mode, all the enantiomers (except for venlafaxine) were baseline separated in resolutions of 4.49, 1.68, 1.88, 1.57, 2.52, 2.33, 3.24, 1.63 and 3.90 for zopiclone, chlorphenamine maleate, brompheniramine maleate, dioxopromethazine hydrochloride, carvedilol, homatropine hydrobromide, homatropine methylbromide, venlafaxine, sibutramine hydrochloride and terbutaline sulfate, respectively. Further, the possible separation mechanism involved was discussed.

## Introduction

The development of chiral separation methods increased vastly during the past decades for the fact that enantiomers have different biological, physiological and pharmacological behaviors on human body. Usually, only eutomer has the desired effect, whereas the uneutomer has unwanted effects such as side effects or toxic effecs [1–3]. In order to separate racemic mixtures, various analytical methods have been developed for chiral separation including HPLC [4–5], GC [6–7] and CE [8–10]. Recently, capillary electrochromatography (CEC), which exploits both the high efficiency of CE and the high selectivity of liquid chromatography stationary phase, emerged as a fairly novel enantiomeric separation technique [11–12]. In general, CEC experiments can be performed with particle packed capillaries, monolithic capillary columns, and open-tubular capillaries (OT-CEC) [13]. In the case of OT-CEC, the chiral selector is coated onto the inner wall of the capillary as stationary phase. OT-CEC is viewed as a promising approach to avoid various problems associated with packed column CEC, such as the difficulties of end-frit fabrication and obtaining uniformly packed columns. Its evident disadvantage, however, is the low separation capability that arises from the low phase ratio on the limited surface area [14–17].

Recently, the use of gold nanoparticles as supports in OT-CEC has aroused the interest of analytical chemists because the gold nanoparticles have larger surface area to enhance the phase ratio [18–20]. O’mahony et al prepared OT-CEC by immobilisation of dodecanethiol gold nanoparticles on prederivatised fused-silica capillaries and applied the columns to separate pyrethroid pesticides [21]. Liu and co-workers described the preparation of OT-CEC columns through the self-assembly of alkanethiols onto AuNP-coated capillaries and investigated their use for the separation of neutral steroid drugs [22]. Besides, *β*-cyclodextrin-modified GNPs were immobilized onto the inner wall of the fused-silica capillaries and applied as special stationary phases for OT-CEC to separate polyaromatic hydrocarbons and three drug enantiomers [23].

**Fig 1:**
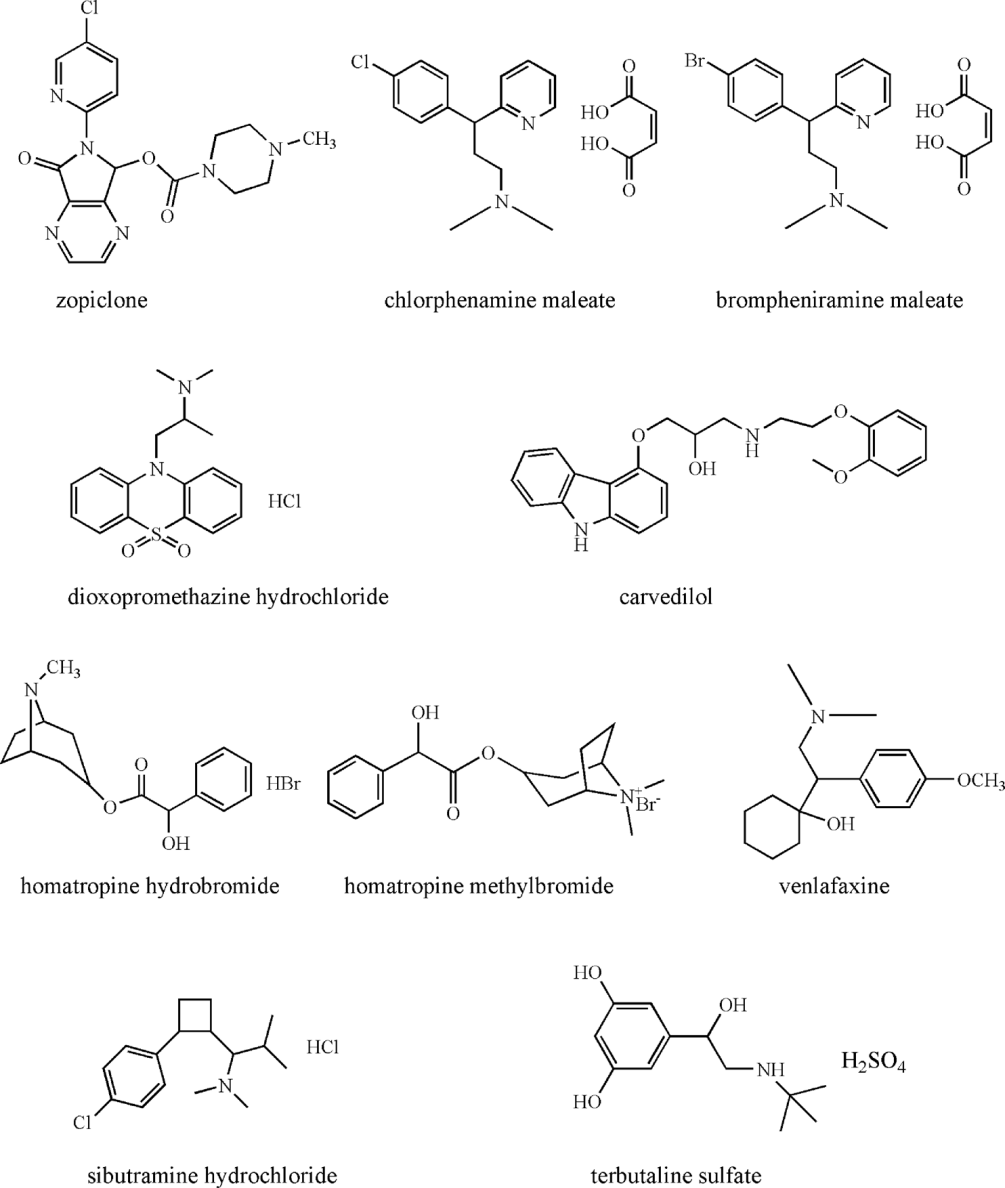
The chemical structures of the ten chiral analytes.

Herein, we first present the preparation of an OT-CEC column by chemically immobilized *β*-cyclodextrin modified gold nanoparticles (*β*-CD-GNPs) onto new surface of prederivatised fused-silica capillary. Then, the work was focused on the combined use of the chiral OT-CEC column and *β*-CD as background electrolyte (BGE) additive in CEC for the enantiomeric separations of (1) zopiclone, (2) chlorphenamine maleate, (3) brompheniramine maleate, (4) dioxopromethazine hydrochloride, (5) carvedilol, (6) homatropine hydrobromide, (7) homatropine methylbromide, (8) venlafaxine, (9) sibutramine hydrochloride and (10) terbutaline sulfate. The chemical structures of the 10 chiral analytes are shown in Fig 1. The influences of BGE pH and the concentration of *β*-CD additive on the enantioseparation efficiency were examined. Finally, the possible separation mechanism was discussed.

## Materials and Methods

### Materials

*(RS)*-Zopiclone, *(RS)*-chlorphenamine maleate, *(RS)*-dioxopromethazine hydrochloride, *(RS)*-carvedilol, *(RS)*-homatropine hydrobromide, *(RS)*-homatropine methylbromide, *(RS)*-venlafaxine, *(RS)*-sibutramine hydrochloride and *(RS)-*terbutaline sulfate were purchased from the National Institute for the Control of Pharmaceutical and Biological Products (Beijing, China). *(RS)*-brompheniramine maleate was obtained from Sigma Chemical Co. (St. Louis, MO, USA). *β*-Cyclodextrin (*β*-CD) was purchased from Bodi Chemical Holding Co., Ltd. (Tianjin, China). *β*-CD-SH was obtained from Shandong Binzhou Zhiyuan Bio-technology Co., Ltd. (Shandong, China). Potassium tetrachloroaurate (III) and (3-mercaptopropyl) trimethoxysilane (MPTMS) were purchased from Shanghai Energy Technology Co., Ltd. (Shanghai, China). Analytical grade sodium hydroxide, phosphoric acid and thiourea were obtained from Shenyang Chemical Regent Factory Co., Ltd. (Shenyang, China). Trisodium citrate dihydrate and tris (hydroxymethyl) aminomethane (Tris) were supplied by Bodi Chemical Holding Co., Ltd. (Tianjin, China).

### Apparatus

All analyses were performed on an HPCE apparatus (CL1030, Beijing Huayang Limin Instrumental Co., Beijing, China), equipped with a power supply, a HW-2000 chromatography workstation and a UV-visible detector. The uncoated fused-silica capillary column (Yongnian Ruifeng Chromatographic Device Co. Ltd, Hebei, China) with a total length of 49 cm (effective length 40 cm to detector) × 50 μm i.d. was used for modification. A modified fused-silica capillary column was used for separations throughout the experiments. UV-visible (UV) and FT-IR (IR) spectra of the synthesised GNPs were recorded with a Shimadzu UV-2201 scanning electron microscopy (SEM) and FT-IR Spectrometer Paragon 1000, respectively. Transmission electron microscopy (TEM) was performed using a JEM 1200 EX TEMSCAN, and SEM pictures of capillaries were obtained using a Hitachi S4000 SEM microscope. The oven used for modifying the capillary was a component of a gas chromatograph 7890A series (Agilent Technologies, Santa Clara, CA, USA).

Each day, the modified capillary was rinsed successively with 0.1mol/L NaOH for 15 min, water for 10 min, and running BGE for 10 min. Between each injection, it was purged with 0.1mol/L NaOH for 2 min, water for 2 min, running BGE for 2 min. An applied voltage 20 kV was used for optimization of the resolution during the separation. Samples were injected into the capillary by hydrodynamic flow at a height differential of 10 cm for 5s. Thiourea was used as a neutral marker to determine the electroosmotic flow (EOF).

A concentration of 25 mM Tris-H_3_PO_4_ was used as BGE buffer and the desired pH was adjusted with 1.0 mol/L H_3_PO_4_. Sample stock solutions were prepared in methanol at 1.0 mg/mL and were stored in a refrigerator. The stock solutions were further diluted into 0.1 mg/mL with distilled water. All the solutions were filtered with a pore size of 0.22 μm filter membranes.

### Preparation of OT-CEC column

**Fig 2:**
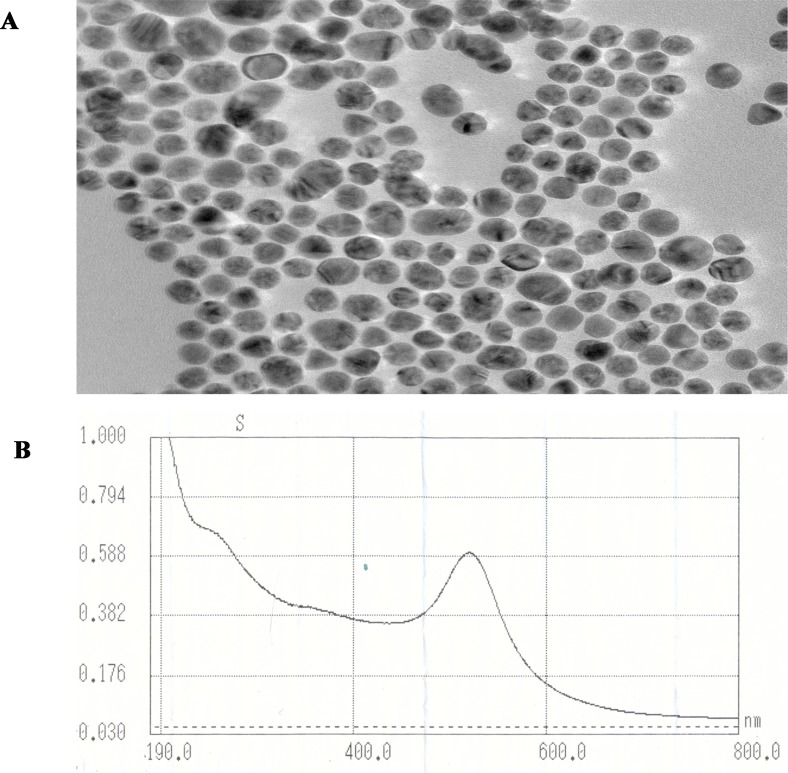
(A) TEM micrograph of gold nanoparticles dispersed in hexane, on a carbon film 400-mesh copper grid; (B) Visible absorption spectrum of gold nanoparticles dispersed in hexane. λ_max_ = 519 nm.

Prior to prepare OT-CEC column, the gold nanoparticles was synthesized according to to published procedure [24]. The synthesized GNPs dispersed in hexane were characterized by TEM and UV-visible spectroscopy (Fig 2A and 2B), respectively. The TEM image (200 kV × 120.0 K magnification) showed the dispersed phase of the GNPs in hexane consisted of spherical particles with an average size of 13 nm (2–3 nm size range). The UV-Vis spectrum of GNPs exhibited plasmon resonance absorption at 519 nm which further confirmed their nanoparticles diameter.

The preparation of capillary column coated with a monolayer of *β*-CD-GNPs was proposed by a method described by O’Mahony [21] with some modification. A capillary column of 49 cm in length was firstly placed in the Beckman P/ACE MDQ capillary electrophoresis system (Fullerton, CA, USA) and then washed by 1.0 M of NaOH under a pressure rinsing (20 psi) for 1 h at 25°C followed by deionized water for 10 min. Upon rinsing with acetone for 30 min, the capillary column was dried in the oven under nitrogen at 60°C for 2 h. After removing acetone, MPTMS solution prepared in ethanol(1%) was then pumped (20 psi) through the capillary under 25°C for 1 h and allowed it to stand overnight. The capillary column was sealed and annealed at 100°C in the oven for 24 h. The synthesized GNPs solution (in water) was pumped (20 psi) through the column at 25°C for 1 h and left to stand for 1 h. The excess GNPs were removed from the capillary with distilled water. Then, the capillary column was flushed with ethanol at 20 psi for 1 h and then allowed to stand for 1 h. After that, a solution of 2 mg/mL of *β*-CD-SH in 50% ethanol solution was pumped through the capillary column for 1 h at 25°C by the pressure of 20 psi and then left to stand for 1 h. Finally, the excess *β*-CD-SH solution was removed from the capillary column by pumping with 50% ethanol and then flushing with ethanol.

**Fig 3:**
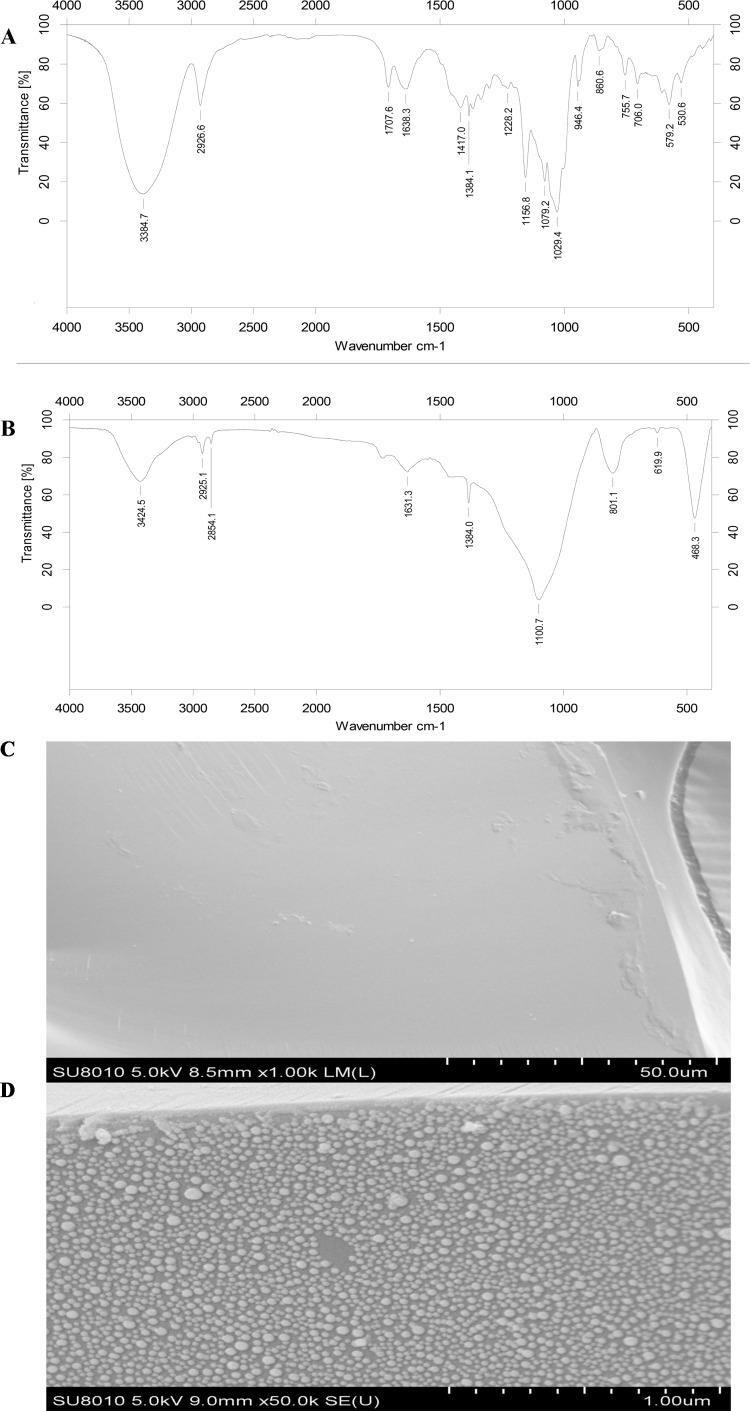
FT-IR spectra of (A) free SH-*β*-CD and (B) *β*-CD-GNPs based capillary; SEM photographs of (C) bare capillary and (D) etched *β*-CD-GNPs capillary.

To investigate whether the *β*-CD-GNPs have been successfully attached onto the capillary, the typical transmission FT-IR spectrums of SH-*β*-CD and *β*-CD-GNPs modified OT-CEC column were compared. As shown in Fig 3A and 3B, strong peaks at 3300–3500 cm^-1^_(νOH)_ and 2920 cm^-1^_(νCH)_ are attributed to the O-H and C-H stretching vibrations of CD. The typical and weak S-H stretching band at 2500–2600 cm^-1^ shown in Fig 3A preliminary proved the presence of *β*-CD-SH, while this peak disappeared in the event of the formation of Au-S bond described in Fig 3B. To further prove the existence of *β*-CD-GNPs etched to the bare capillary, the SEM image of bare capillary shown in Fig 3C (5 kV × 1.0 K magnification) and etched *β*-CD-GNPs capillary shown in Fig 3D (5 kV × 50.0 K magnification) were also determined. Fig 3C showed a smooth capillary wall, yet a rough capillary wall was obtained in Fig 3D, indicating that the coverage of *β*-CD-GNPs on the column wall is quite extensive. The above results guarantee that *β*-CD-GNPs have been successfully attached onto the surface of capillary column.

**Fig 4:**
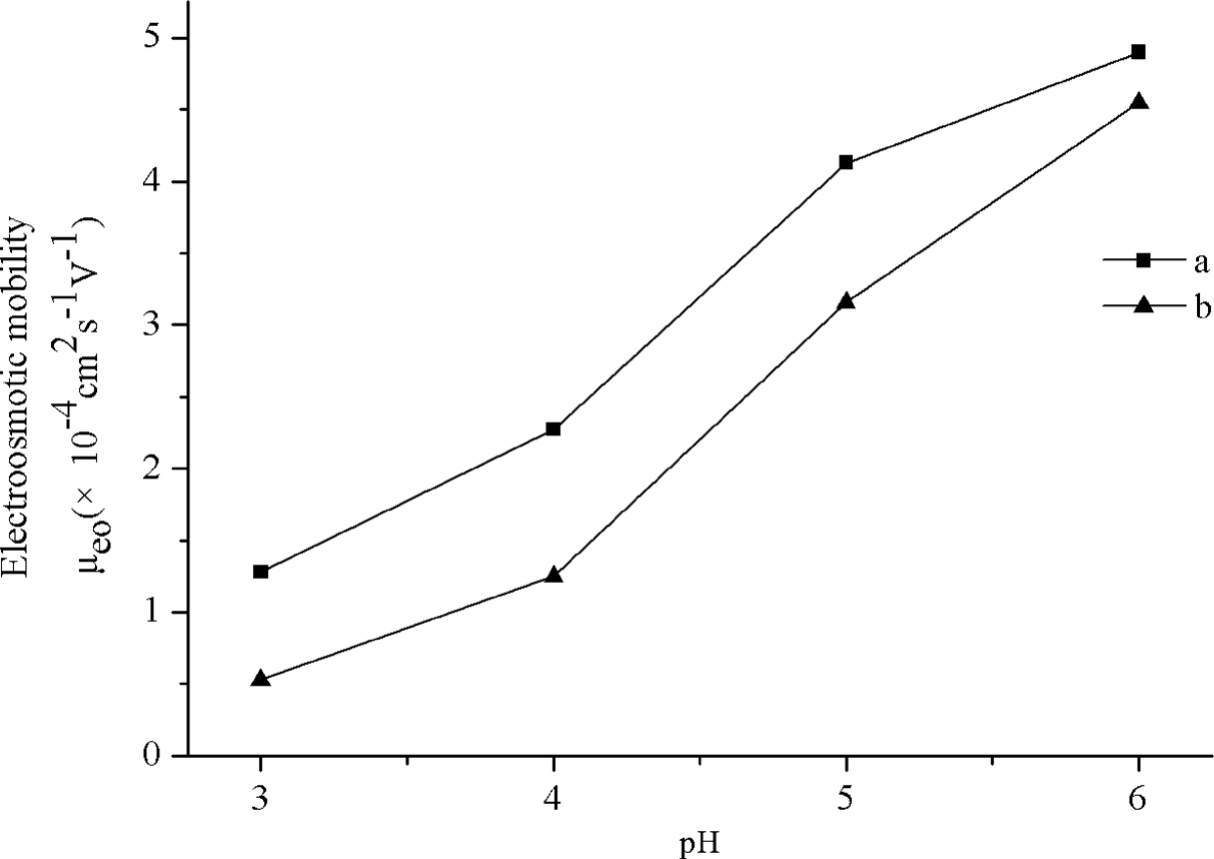
The effect of pH on the EOF mobility. BGE: pH 2.5, 25 mM Tris-H_3_PO_4_ buffer solution, 20kV. (a) blank capillary; (b) *β*-CD-GNPs based capillary. OTCEC conditions: 49 cm × 50 μm i.d., Sample: thiourea, Separation voltage, 20 kV.

EOF flow is the main driving force in capillary electrophoresis. In the present study, we investigated the EOF value of the bare capillary in the varied pH from 3.0 to 6.0 (other conditons: 25 mM Tris-H_3_PO_4_ buffer solution, 20 kV). From Fig 4(A), it can be seen that EOF mobility of bare capillary increase greatly with the increase of pH from 3.0 to 6.0 which is generated by electrical double layer on the inner surface determined by its surface charge density. Meanwhile, as shown in Fig 4(B), under the same buffer conditions, *β*-CD-GNPs based capillary displays a similar mobility profile to that of an untreated capillary and as expected the *μ*_eo_ values of the modified capillary was lower than the bare capillary due to silanol blocking.

## Results and Discussion

### Enantioseparation of ten basic drugs

In our preliminary work, the separation conditions of the 10 drug enantiomers, zopiclone, chlorphenamine maleate, brompheniramine maleate, dioxopromethazine hydrochloride, carvedilol, homatropine hydrobromide, homatropine methylbromide, venlafaxine, sibutramine hydrochloride and terbutaline sulfate were optimized on the OT-CEC column. Tris-H_3_PO_4_ buffer with concentration of 25 mM and pH varying from 2.0 to 3.0 was used as the running buffer. It was found that the migration time of all analytes decreased as the pH increased, mainly due to increased electroosmotic mobility (EOF). However, no chiral recognition was observed for any of the studied basic analytes. In order to improve the efficiency of separation, *β*-CD (5.0 mM) was added to the BGE as chiral additive. As expected, significantly improvement in enanotiomeric separation was achieved. In this case, the effects of BGE pH and added CD concentration on the enantioseparation were studied.

**Fig 5:**
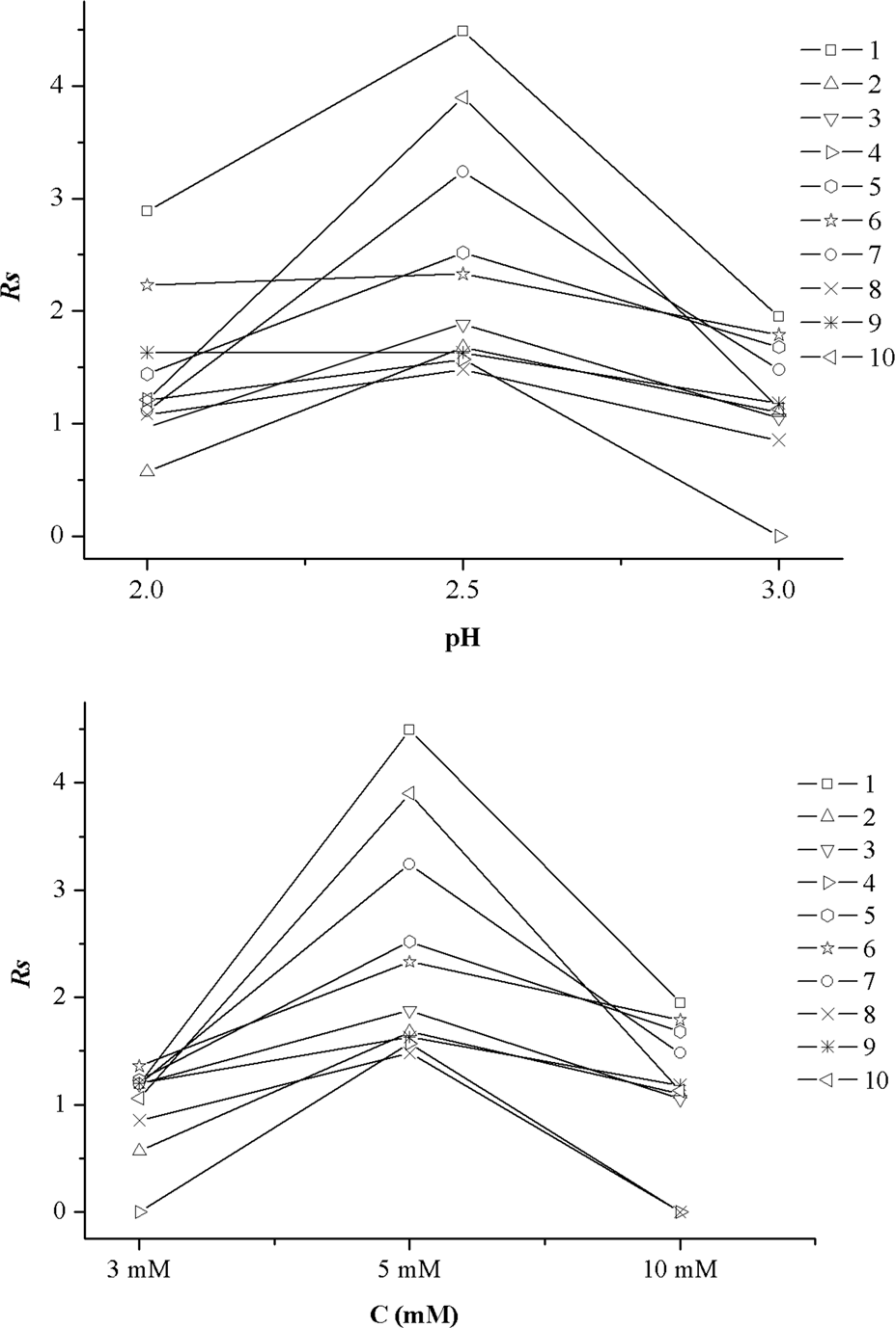
Effect of pH (A) and *β*-CD concentration (B) on *R*_*S*_ of the 10 chiral analytes (1) zopiclone, (2) chlorphenamine maleate, (3) brompheniramine maleate, (4) dioxopromethazine hydrochloride, (5) carvedilol, (6) homatropine hydrobromide, (7) homatropine methylbromide, (8) venlafaxine, (9) sibutramine hydrochloride and (10) terbutaline sulfate.

The effect of the BGE pH on the enantioseparation was investigated over the range of 2.0–3.0 using 25 mM Tris-H_3_PO_4_ buffer containing 5.0 mM *β*-CD at an applied voltage of 20 kV. As can be seen in Fig 5A, the chiral resolutions of studied drugs apparently increased with increasing the pH range from 2.0 to 2.5, then decreased at a pH 3.0 (except for sibutramine hydrochloride). This decreased resolution might be due to the decreased interaction opportunity between chiral selector and analyte resulting from the enhancement of EOF at pH 3.0. Therefore, pH 2.5 was chosen as the optimized pH value for the chiral separation of the studied drugs (for sibutramine hydrochloride, pH 2.0 was selected).

To investigate the influence of the *β*-CD concentration on the resolutions of the ten drugs, the concentration of *β*-CD was varied from 3.0 to 10.0 mM when the other conditions were as follows: 25 mM Tris-H_3_PO_4_ buffer at pH 2.5 (sibutramine hydrochloride, pH 2.0) and operation voltage of 20 kV. Fig 5B demonstrates the *Rs* values of the ten analytes at each CD concentration. An increase in *Rs* for most drug enantiomers was observed from 3.0 mM to 5.0 mM *β*-CD concentration due to an increase of the interactions between *β*-CD and analytes. However, when the concentration of CD became larger than 5 mM, the *Rs* of these enantiomers decreased obviously. It seemed that the analytes were probably fully complexed at CD concentration of 5 mM, and a further increase in concentration had negative effects on *Rs*.

**Fig 6:**
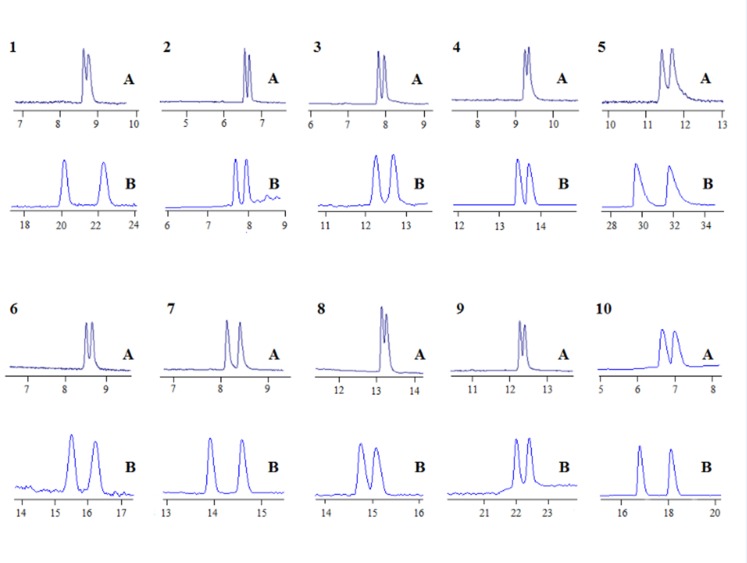
Typical electropherograms of the 10 chiral analytes on (A) bare capillary and (B) etched capillary. BGE: 20 kV, (A) 10 mM *β*-CD, pH 2.5, 30 mM Tris-H_3_PO_4_, (B) 5 mM *β*-CD, pH 2.5 (sibutramine hydrochloride, pH 2.0), 25 mM Tris-H_3_PO_4_. (the numbers of the drugs are the same as in **Fig 5**).

In summary, the optimal BGE for the separation of the analytes was 25 mM Tris-H_3_PO_4_ and 5 mM *β*-CD at pH 2.5 (sibutramine hchloride, pH 2.0) at a voltage of 20 kV. Under such separation conditions, all the analytes (except for venlafaxine) were baseline separated (Fig 6B). For zopiclone, chlorphenamine maleate, brompheniramine maleate, dioxopromethazine hydrochloride, carvedilol, homatropine hydrobromide, homatropine methylbromide, venlafaxine, sibutramine hydrochloride and terbutaline sulfate, the separation resolutions (averaged over three runs) were: 4.49, 1.68, 1.88, 1.57, 2.52, 2.33, 3.24, 1.48, 1.63, 3.90, respectively. The electrochromatograms obtained under optimized separation conditions are shown in Fig 6B.

To test the run-to-run reproducibility, six consecutive separations of two pairs of the drug enantiomers (zopiclone and homatropine methylbromide) were performed. The RSDs of the retention time was lower than 2.5% for two drug enantiomers. The reproducibility of the three freshly prepared chiral OT-CEC columns was also investigated in terms of retention time with zopiclone as model solute. The RSD of the retention time was lower than 4.3%, indicating good column to column reproducibility of three chiral column. In addition, these capillaries appeared to be stable for up to one month when not in use and stored in distilled H_2_O.

BGE (A): 5 mM *β*-CD, 25 mM Tris-H_3_PO_4_, 20 kV; BGE (B): pH 2.5 (sibutramine hydrochloride, pH 2.0), 25 mM Tris-H_3_PO_4_, 20 kV.

### Discussion of separation mechanism

**Fig 7:**
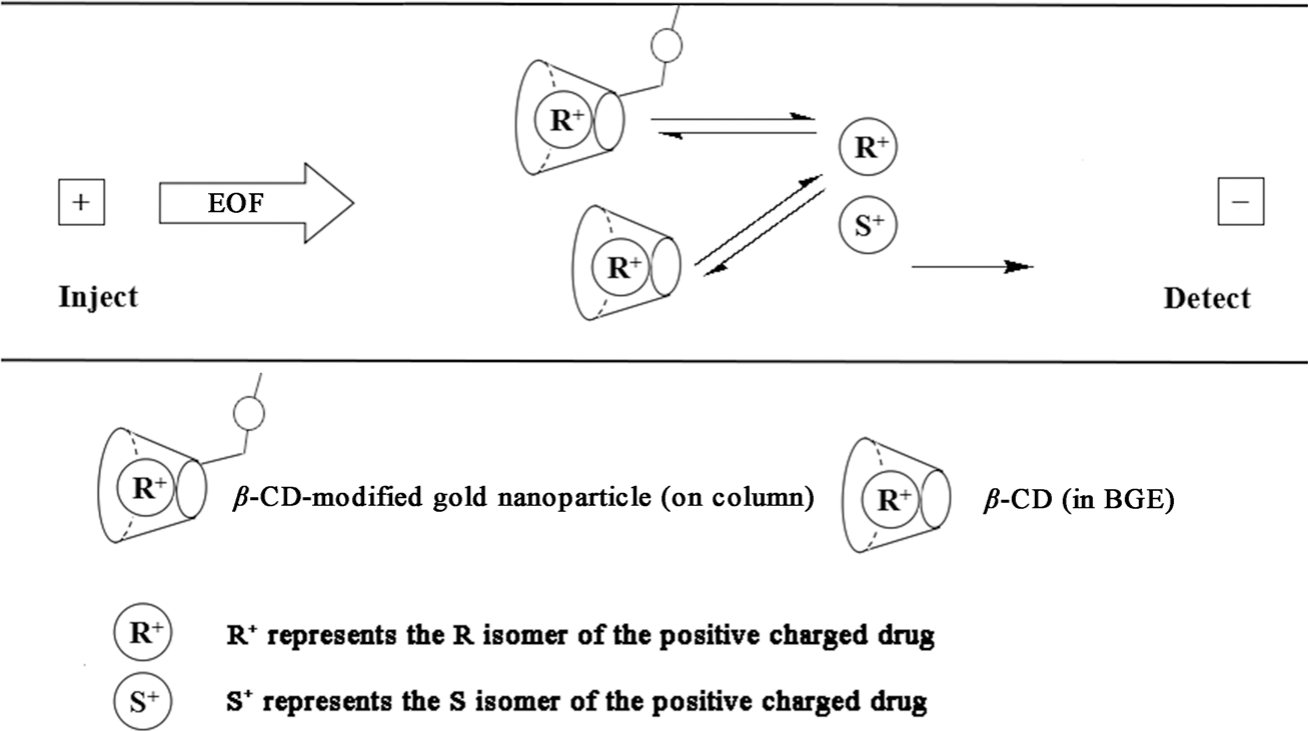
Illustration of chiral separation of positively charged analytes on *β*-CD-GNPs modified capillary.

As mentioned above, when only OT-CEC column was utilized in the enantioseparation of the ten chiral drugs, no resolution was observed. However, OT-CEC column accompanying with a BGE containing *β*-CD could enantioseparate the ten chiral drugs very well. Fig 6(A) shows the electrophorogram of separation of the ten drug enantiomers when *β*-CD was used as chiral addictive by capillary zone electrophoresis. Under our opitimized separation conditons, nine pairs of drug enantiomers were partially enantioseparated, with the *R*s values of 0.78, 1.16, 1.30, 1.28, 1.12, 1.21, 0.76, 0.92, 1.10 for zopiclone, chlorphenamine maleate, brompheniramine maleate, dioxopromethazine hydrochloride, carvedilol, homatropine hydrobromide, venlafaxine, sibutramine hydrochloride and terbutaline sulfate, respectively. Only homatropine methylbromide was baseline resolved with *R*s of 1.97. Compared with Fig 6(A) and seen in Fig 6(B), it can be seen obviously that when an OT-CEC column was utilized, the resolution of all the 10 chiral drugs was improved greatly from partial separation to complete separation, indicating that the CD on the OT-CEC column had a greater contribution in enantioseparation of the 10 chiral drugs. It was thus clear that there was a synergistic effect between *β*-CD in BGE and on the OT-CEC column for the enantioseparation. Fig 7 illustrates the enantioseparation mechanism by OT-CEC column. The analyte enantiomers could interact with the CD on the chiral column. If the *R*-enantiomer had a greater affinity with the CD on the column, the migration time of the *R*-enantiomer should be longer than the *S*-enantiomer. At the same time, if the *R*-enantiomer had a greater affinity with the CD in the BGE, the electrophoretic mobility of the *R*-enantiomer should be smaller due to its smaller mass charge ratio, resulting in a longer migration time of *R*-enantiomer. Therefore, the synergistic effect occurred and improved the separation efficiency.

## Conclusions

In this paper, we described the development of a novel chiral OT-CEC column, which was prepared by the chemically immobilized *β*-CD-GNPs on prederivatised MPTMS capillary. Successful enantioseparation of ten racemic drugs was achieved on the CEC column by combining the use *β*-CD as BGE additive. Comparing to the results obtained in capillary electrophoresis with a comparable amount of *β*-CD as chiral selector alone, we got the enhancement of enantioseparation of ten drug enantiomers by using *β*-CD modified OT-CEC column in capillary electrochromatography. The study demonstrated that there was a synergistic effect between *β*-CD in BGE and on the OT-CEC column for the enantioseparation.
